# Elder abuse and life-course victimization in hospitalized older adults in Sweden: prevalence and associations with mental ill-health

**DOI:** 10.1186/s12877-022-03638-8

**Published:** 2022-12-02

**Authors:** Nicolina Wiklund, Mikael Ludvigsson, Katarina Nägga, Johanna Simmons

**Affiliations:** 1 Department of Acute Internal Medicine and Geriatrics in Linköping and Department of Health, Medicine and Caring Sciences, Linköping University, Linköping, Sweden; 2 Department of Geriatrics, Örebro University Hospital, Örebro, Sweden; 3 Department of Psychiatry in Linköping and Department of Biomedical and Clinical Sciences, Linköping University, Linköping, Sweden

**Keywords:** Cumulative inequality, Interpersonal violence, Life-course perspective, Polyvictimization

## Abstract

**Background:**

The prevalence of elder abuse has only rarely been investigated in Sweden and never in a hospital setting. Therefore, the aims of this study were to: 1) Estimate the prevalence of elder abuse and life-course victimization among hospitalized older adults in Sweden, 2) Explore factors associated with elder abuse in the same sample, and 3) Explore the associations between life-course victimization and mental ill-health.

**Methods:**

The study was conducted at a university hospital in Sweden. Adults over the age of 65 years admitted to a medical or geriatric acute care ward during spring 2018 were consecutively recruited. The participant rate was 44% (*n* = 135/306). Participants were assessed via a face-to-face interview about their experiences of elder abuse and abuse earlier in life. Mental ill-health was measured using a self-administered depression assessment (Patient Health Questionnaire-9), along with information about medications and diagnoses retrieved from medical records.

**Results:**

Altogether, 40.7% (*n* = 55) of the participants reported some form of abusive experience during their life course. The prevalence of elder abuse was 17.8% (*n* = 24), and 58% (*n* = 14) of elder abuse victims also reported victimization earlier in life. Being abused before the age of 65 was the only background factor associated with elder abuse (OR = 5.4; 95% CI 1.9–15.7). Reporting abusive experiences both before and after the age of 65 was associated with current anti-depressant medication (OR = 6.6; 95% CI 1.1–39.2), a PHQ-9 result of 10 or more (OR = 10.4; 95% CI 2.1–51.0), and nine or more symptom diagnoses (OR = 4.0, 95% CI 1.0–16.1). Being abused only before or after the age of 65 was not significantly associated with any mental ill-health outcome measure.

**Conclusions:**

Elder abuse and victimization earlier in life are highly prevalent among hospitalized older patients, and our findings underline the importance of a life-course perspective both in research on elder abuse and in clinical practice. Identifying and caring for older adults who have been subjected to abuse should be a priority in health care.

**Supplementary Information:**

The online version contains supplementary material available at 10.1186/s12877-022-03638-8.

## Background

Elder abuse is recognized as a serious public health problem [1], and includes neglect as well as physical, emotional, sexual, and financial abuse. It can be perpetrated by professional caregivers, family members, or other persons in a position of trust [2]. Exposure to abuse in later life has been associated with various poor health outcomes, including depression, anxiety, and suicidality, as well as increased health care consumption and higher mortality [3, 4].

The prevalence of elder abuse is estimated at 10–16% in community-dwelling populations worldwide [5, 6]. Few studies reporting the prevalence of elder abuse in Sweden have been published, and there is a wide variation in results, i.e., between 3 and 31% [7–9]. The large discrepancy between studies can most likely be attributed to methodological differences concerning aspects such as definition and operationalization of elder abuse, the population studied, and characteristics of respondents [10, 11]. Research into the prevalence of elder abuse is often carried out using questionnaires or structured telephone surveys. When using these types of survey-based methods in a community setting, there is a high risk of non-participation from individuals with high morbidity or low ADL (activities of daily life) functioning [12, 13]. It is known that elder abuse is associated with increased functional dependence and higher morbidity [8, 14–16]. Hence, there is a risk that the older adults at the greatest risk of elder abuse will remain unheard in population-based studies. At the other end of the spectrum are studies conducted in long-term care settings which often include older adults with high functional dependency, but often rely on third-party reports. No prevalence study has been conducted in in long-term care in Sweden and they are scarce internationally. However, a global meta-analysis found that 64% of staff working in institutional settings admitted to perpetrating elder abuse in the past year [17]. The high prevalence is likely explained in part by a higher prevalence of the previously mentioned risk factors for elder abuse (morbidity, ADL functioning) among residents in long-term care facilities compared to community-dwelling older adults. Also, there is a possible bias when using proxy reports instead of retrieving information from the older adults themselves.

Considering that elder abuse is a serious health problem and that elder abuse victims are often in contact with health care providers, detecting elder abuse in the health care system has been recommended [18]. Sweden has no laws about mandatory reporting of elder abuse, but the National Board of Health and Welfare has issued a recommendation that health care professionals should ask all patients (regardless of age) questions about violence whenever there are signs or symptoms of this [19]. Still, one Swedish study found that although 25% of older adults (aged ≥ 65 years) reported life-time experiences of physical, emotional, or sexual abuse, only 2% of all respondents had been asked questions within health care about victimization [20]. This finding indicates that older adults’ experiences of abuse often remain hidden in health care encounters and illustrates the importance of further investigating various aspects of elder abuse in a hospital setting, including the prevalence of abusive experiences among patients. The prevalence of elder abuse in a hospital setting has thus far been inadequately examined and cannot be directly compared to the prevalence reported in either population-based surveys or studies in long-term care settings. It is likely that a hospital-based population will include a larger proportion of older adults, who – due to functional dependency issues – are at a greater risk of elder abuse, and who are less likely to participate in general population studies. One previous study, conducted on a hospital ward in India, estimated an elder abuse prevalence of 16% [21]. The prevalence of elder neglect – one type of elder abuse – has been examined in a hospitalized population in Israel, where a prevalence rate of 14% was found [22]. The small number of studies is a limitation, which indicates the need for further prevalence studies among this population.

Studies of background factors associated with elder abuse should ideally also be examined in different settings and populations. Most previous research has been conducted among community-dwelling populations [5, 17]. Alongside morbidity and low ADL functioning, cognitive impairment, low education level, and low social support have also been suggested as factors associated with elder abuse [8, 14–16]. The research on associated factors in other settings is limited, but one example is a study that investigated abuse by paid caregivers in long-term care [23]. In that study, an association between abuse and low ADL functioning was found as well as between abuse and behavioral problems among care recipients, e.g., being verbally or physically abusive or actively resisting care. Indicators of cognitive impairment was however not significantly associated with abuse when also considering behavioral problems in analyses [23].

Studies about elder abuse often investigate prevalence and associated ill-health in the past 12 months. However, there is increasing support from research that childhood abuse is associated with elder abuse [24–26] as well as with poor health outcomes in later life [27–29]. It is therefore important to consider elder abuse from a life-course perspective. One theory based on the life-course perspective is the cumulative inequality theory [30, 31], which attempts to explain how an individual’s life trajectory develops during the life course, and how inequality and disadvantages in different areas of life are self-reinforcing and cumulate in a systematic way. It is possible that childhood abuse, elder abuse, and poor health outcomes are all part of the same negative life trajectory. For example, it has been theorized – with the support of cumulative inequality theory – that childhood adversities can cause elder abuse, mediated by decreased physical and mental health in middle age [32]. Related to this, studies on childhood abuse have found that polyvictimization, i.e., being subjected to multiple forms of abuse, is more strongly associated with repeat victimization and poor health than any single form of abuse [33–35]. Polyvictimization has only recently been considered in studies on elder abuse, but is reported to be highly prevalent and more strongly associated with physical and mental ill-health than single victimization [14, 36, 37].

Altogether, the prevalence of elder abuse among hospitalized older adults has never been reported in Sweden, and only rarely internationally. Also, the life-course perspective is often lacking in research on elder abuse and associated ill-health. Therefore, using a hospitalized sample of older adults in Sweden, our aims were:

- 1. To estimate the prevalence of a) elder abuse and b) life-course victimization.
- 2. To investigate if experiences of abuse before the age of 65 were associated with elder abuse.
- 3. To investigate associations between life-course experiences of abuse and mental ill-health.

## Methods

### Procedure

This study was part of the larger Responding to Elder Abuse in GERiAtric care (REAGERA) study conducted in Sweden. Data was collected during spring 2018 at a university hospital clinic with one acute medical ward and one acute geriatric ward. Common reasons for admission included pneumonia, congestive heart failure, and acute exacerbation of chronic obstructive lung disease. Patients over the age of 65 years admitted during the study period were eligible for inclusion and were recruited consecutively on weekdays. The exclusion criterion was insufficient physical, cognitive, or linguistic capacity to participate, which was subjectively assessed by the nurse on the ward. The main purpose of the data collection was to validate a screening instrument (the REAGERA-S, where S stands for self-administered) [38]. Participants first completed the REAGERA-S [38], along with follow-up questions, including PHQ-9 (Patient Health Questionnaire-9). Thereafter, a semi-structured interview was conducted with the help of an interview guide to evaluate exposure to abuse (supplementary file 1). Whenever experiences of abuse were revealed, qualitative interviews were conducted covering both the experiences of elder abuse [39] and how abusive experiences were managed by the older adults [40].

### Sample size

As previously mentioned, this study is based on a secondary analysis of the data collected to validate the REAGERA-S [38]. Sample size was therefore estimated using a sensitivity and specificity analysis for screening and diagnostic tests, as recommended by Bujang and Adnan [41]. Using an estimated prevalence of elder abuse of 10%, a sensitivity of the instrument of 90%, and a null hypothesis of 50%, a sample size of 120 participants was required for the validation study [41].

### Measures

#### Victimization

During the interviews, which were carried out by authors NW, ML, or JS, an assessment was made of whether the participant had been exposed to abuse and, if so, when in life it had occurred. Elder abuse was defined as an abusive experience that occurred after the age of 65. All five forms of elder abuse (physical, emotional, sexual, financial, or neglect) were considered, as was abuse by different kinds of perpetrators (partner, family member, care provider, or other). However, because some forms of abuse were experienced by only very few participants, separate prevalence rates for the different forms of abuse are not presented. In the case of uncertainty about whether to classify an experience as abusive, the ambiguity was discussed among the interviewers before classification. The interviews with victims of elder abuse were also recorded and transcribed for the purpose of subsequent qualitative studies about experiences of abuse [39, 40]. Hence, when necessary, the transcripts could be used to reach a consensus agreement on abuse classification.

In total, four different variables measuring abusive experiences were created: 1) Any life-course experiences of abuse, regardless of age: yes or no. 2) Abuse ≥ 65 years (elder abuse): yes or no. 3) Abuse < 65 years (earlier in life abuse): yes or no. 4) “Age at abuse”, one variable with four mutually exclusive categories: A) No abuse, B) Abuse only < 65 years, C) Abuse only ≥ 65 years, and D) Abuse both before and after the age of 65.

#### Background information and covariates

Data about age and sex was collected from medical records. Age was categorized into three groups: 65–74 years (young old), 75–84 years (middle old), and > 85 years (oldest old). Sex was categorized as either male or female. Information about activities of daily living (ADL) and education levels was collected during the interview (supplementary file 1). The ADL questions were condensed into one ordinal variable with three levels: needing no assistance, needing assistance with instrumental ADL (e.g., medications, cleaning), or needing assistance with both instrumental ADL and basic ADL (e.g., personal hygiene). Education level was categorized into three different groups: nine or fewer years of schooling (corresponding to elementary school), 10–12 years (corresponding to secondary school), or 13 years or more (corresponding to higher education). Marital status was categorized as married or living together under marital circumstances, or non-married. Non-married also included divorcees and widows/widowers.

#### Mental ill-health measures

Mental ill-health was measured in four different ways: 1) A self-administered screening instrument (Patient Health Questionnaire-9, PHQ-9), 2) Having depression or anxiety listed as a diagnosis in the medical records, 3) Current medication with an anti-depressant medication, or 4) Having nine or more symptom diagnoses according to medical records.

PHQ-9 is a nine-item questionnaire used to screen for depression in all age categories. With a score of 10 or higher (maximum 30), both sensitivity and specificity for detecting major depression have been estimated at 88% [42]. Internal reliability of the scale, as measured by Cronbach’s alpha, was previously reported at 0.86–0.89 in different samples [42], and was also satisfactory in this sample (Cronbach’s alpha = 0.8). Although there is no general agreement, Cronbach’s alpha of > 0.7 is often considered as acceptable [43]. The PHQ-9 was independently filled out by the participant before the interview.

Information about diagnoses and medications was gathered from medical records. The county in which the study was conducted began using the current system for medical records in 2008, and the diagnoses were therefore limited to the past ten years. The review of diagnoses and medications was blinded, as it was conducted by one of the authors who did not conduct the interviews and therefore did not know the status of abuse exposure. The diagnostic classification system used was ICD-10, and anxiety coded as F41 and depression coded as F32–F33 were included. Because none of the participants reporting abuse only after the age of 65 had an anxiety diagnosis, data on anxiety and depression was computed into one variable, separating between participants with or without a depression or anxiety diagnosis. Data on medications was collected from the current medication list. Anti-depressant medication was defined as selective serotonin reuptake inhibitors (SSRIs), serotonin and norepinephrine reuptake inhibitors (SNRIs), or noradrenaline and specific serotonergic antidepressants (NaSSAs).

The risk of mental ill-health increases with increasing number of somatic symptom diagnoses [44, 45]. An association between depression and somatic symptoms has also been reported in older adults [46]. We therefore used symptom diagnoses as a complementary proxy marker for depression and anxiety in this study. Somatic symptoms were registered using diagnostic codes according to the ICD-10 in the patients’ medical records (R00–R99). These diagnostic codes are used when a patient presents with a symptom that remains medically unexplained. The total number of somatic symptom diagnoses was calculated and dichotomized into those with fewer than nine symptom diagnoses (*n* = 91, 72.8%) and those with nine or more symptom diagnoses (*n* = 34, 27.2%). The cut-off was chosen to create one group including those with the higher number of symptom diagnoses.

### Statistics

The data was analyzed using SPSS version 28. The significance level was set to *p* = 0.05 in all analyses.

#### Aim 1, prevalence of abuse

Descriptive statistics were produced to examine prevalence rates of elder abuse as well as any life-course victimization and age at abuse.

#### Aim 2, association between earlier in life victimization and elder abuse

To investigate if abuse before the age of 65 was associated with elder abuse, a multivariate logistic regression analysis with elder abuse as the dependent variable was performed. Earlier in life abuse (< 65 years) was used as an independent variable and the model also included the following independent variables: sex, age category, ADL level, education level and marital status. Area under the receiver operating curve (ROC) was used to assess the model’s goodness-of-fit.

#### Aim 3, associations between victimization and mental ill-health

First, Pearson’s chi square test was used to test for bivariate associations between potential confounding variables, i.e., background characteristics (sex, age category, ADL level, education level, and marital status) and the different measures of mental ill-health, as well as age at abuse. No significant association was found in the analyses including educational level and that variable was therefore excluded from further analyses. Thereafter four different multivariate logistic regression analyses were performed, using the different measures of mental ill-health as dependent variables and age at abuse as well as sex, age, ADL status, and marital status as independent variables. For all logistic regression models, area under the ROC curve was used to assess the models’ goodness-of-fit.

## Results

**Fig. 1:**
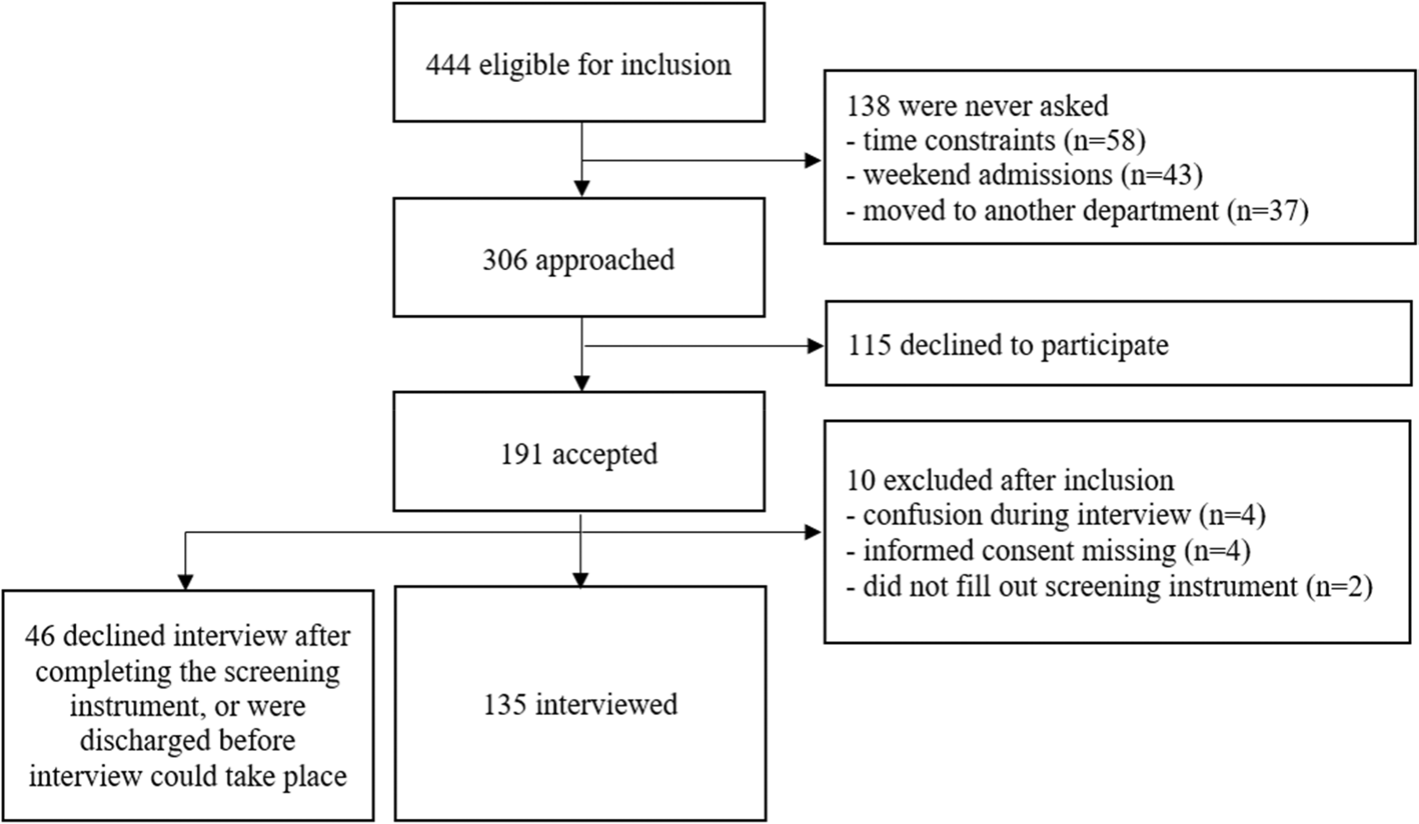
Derivation of the analytical sample from the hospital clinic (*n* = 135)

Figure 1 presents a flowchart for the recruitment of participants. During the inclusion period, 668 older patients were treated at the clinic. 224 were excluded due to insufficient physical (n = 85), cognitive (n = 118), or linguistic (n = 21) capacity to participate, leaving 444 eligible participants who met the inclusion and exclusion criteria. However, 138 were not asked to participate for organizational reasons, as specified in Fig. 1. Altogether, 306 patients were approached, 191 accepted, and 135 patients were interviewed and hence included in the study (participant rate 44%, *n* = 135/306).

### Aim 1. Prevalence of elder abuse and life-course victimization

Altogether, 55 (40.7%) of the participants reported some form of abusive experience during their life course (Table 1). Elder abuse was reported by 24 older adults (17.8%) and 14 participants (58.3% of elder abuse victims, 10.4% of the entire sample) reported abusive experiences both before and after the age of 65.

**Table 1:** Prevalence of life-course victimization and elder abuse (*n* = 135)

	**N**	**%**
**Life-course victimization**		
No abuse	80	59.3
Any life-course victimization	55	40.7
**Elder abuse**		
No elder abuse	111	82.2
Elder abuse (age ≥ 65)	24	17.8
**Age at abuse**		
No abusive experiences	80	59.3
Abuse only < age 65	31	23.0
Abuse only ≥ age 65	10	7.4
Abuse both < and ≥ age 65	14	10.4

### Aim 2. Background factors associated with reporting elder abuse

The background characteristics of the sample are presented in Table 2. Neither sex, age, ADL status, education, nor marital status were associated with elder abuse. Only abuse before 65 years was associated with increased odds of reporting elder abuse (OR = 5.4; 95% CI 1.9–15.7) (Table 2).

**Table 2:** Background characteristics of the total sample, elder abuse victims, and factors associated with elder abuse (*n* = 135)

	**Total sample** **(*n* = 135)**		**Elder abuse victims (*n* = 24)**		**Odds of reporting elder abuse**	
	**n**	**%**	**n**	**%**	**OR**	**95% CI**
**Sex**						
Men	62	45.9	9	37.5	1	
Women	73	54.1	15	62.5	1.0	0.3–2.8
**Age**						
65–74	31	23.1	6	26.1	1	
75–84	55	41.0	7	30.4	0.7	0.2–2.5
≥85	48	35.8	10	43.5	1.3	0.3–5.1
**ADL level**						
No help	62	46.6	8	33.3	1	
I-ADL	46	34.6	10	41.7	1.9	0.6–6.3
B-ADL	25	18.8	6	25.0	1.6	0.4–6.2
**Education level**						
≤9 years	75	56.4	11	45.8	1	
10–12 years	27	20.3	7	29.2	1.8	0.5–5.9
≥13 years	31	23.3	6	25.0	1.3	0.4–4.2
**Marital status**						
Non-married	67	49.6	13	54.2	1	
Married	68	50.4	11	45.8	0.6	0.2–1.8
**Abuse < age 65**						
No	90	66.7	10	41.7	1	
Yes	45	33.3	14	58.3	5.4**	1.9–15.7

*OR* Odds ratio, *CI* Confidence interval. Missing cases in regression analysis *n* = 5 (3.7%). Area under the ROC: 0.75 (95% CI 0.64–0.86)

^**^ = p < 0.01

### Aim 3. Association between life course experiences of abuse and mental ill-health

As can be seen in Table 3, reporting only elder abuse or only abuse before 65 years was not significantly associated with any of the mental-health outcomes. However, reporting abuse both before and after 65 years of age was associated with a PHQ-9 score ≥ 10 (OR = 10.4 95% CI 2.1–51.0), anti-depressant medications (OR = 6.6 95% CI 1.1–39.2), and reporting ≥ 9 symptom diagnoses (OR = 4.0, 95% CI 1.0–16.1). Having a depression or anxiety diagnosis was not significantly associated with any life-course victimization. Female sex was significantly associated with depression or anxiety diagnosis (OR 3.0 95% CI 1.1–8.7), and ADL level was significantly associated with anti-depressant medication (B-ADL: OR 7.8 95% CI 1.3–47.0) as well as having ≥ 9 symptom diagnoses (B-ADL: OR 5.9 95% CI 1.6–21.2, I-ADL: OR 3.7 95% CI 1.2–11.0) Also, being married was significantly associated with having a result ≥ 10 on PHQ-9 (OR 3.2 95% CI 1.0–9.9).

**Table 3:** Multivariate logistic regression models for associations between abuse during the life course and mental ill-health (*n* = 135)

		**PHQ-9 ≥ 10**		**Depression or** **anxiety**		**Anti-depressant medication**		**≥ 9 symptom** **diagnoses**	
	**n**	**OR**	**95% CI**	**OR**	**95% CI**	**OR**	**95% CI**	**OR**	**95% CI**
**Age at abuse**									
No abusive experiences	80	1		1		1		1	
Abuse only < age 65	31	1.9	0.6–6.1	2.2	0.7–6.5	3.3	0.8–14.4	0.8	0.3–2.5
Abuse only ≥ age 65	10	0.9	0.1–10.1	1.0	0.1–9.3	3.6	0.3–44.0	0.5	0.0–4.4
Abuse both < and ≥ age 65	14	10.4**	2.1–51.0	2.8	0.7–11.4	6.6*	1.1–39.2	4.0*	1.0–16.1
**Sex**									
Male	62	1		1		1		1	
Female	73	0.6	0.2–1.7	3.0*	1.1–8.7	3.2	0.7–14.1	3.2*	1.2–8.7
**Age**									
65–74	31	1		1		1		1	
75–84	55	1.7	0.4–6.7	0.5	0.2–1.6	1.4	0.3–6.9	1.0	0.3–3.1
≥85	48	2.3	0.5–10.2	0.5	0.1–2.0	0.5	0.1–3.1	0.8	0.2–2.9
**ADL level**									
No help	62	1		1		1		1	
I-ADL	46	2.0	0.6–6.7	0.8	0.3–2.6	3.8	0.8–17.3	3.7*	1.2–11.0
B-ADL	25	1.1	0.3–4.9	2.4	0.7–8.5	7.8*	1.3–47.0	5.9**	1.6–21.2
**Marital status**									
Non-married	67	1		1		1		1	
Married	68	3.2*	1.0–9.9	0.9	0.4–2.5	2.4	0.6–9.0	1.9	0.7–5.0

Area under the ROC for each respective model: PHQ-9: 0.78 (95% CI 0.68–0.88); Depression or anxiety diagnoses 0.73 (95% CI 0.62–0.83); Anti-depressant medication 0.81 (95% CI 0.71–0.92); Symptom diagnoses 0.76 (95% CI 0.67–0.86). Missing cases PHQ-9 = 31; Depression or anxiety diagnoses = 15; Anti-depressant medication *n* = 16; Symptom diagnoses *n* = 12

*OR* Odds ratio, 95% *CI* 95% confidence interval, *I-ADL* need for support for instrumental ADL, *B-ADL* need for support for basic ADL

^*^ = *p* < 0.05,

^**^ = *p* < 0.01

#### Model fit

The values for area under the ROC curve for each logistic regression model are presented in Tables 2-3 and were found to range between 0.73 and 0.81. Hosmer and Lemeshow suggest that values between 0.7 and 0.8 are acceptable, while 0.8–0.9 is excellent [47], and the fit for all models was hence deemed to be satisfactory.

## Discussion

This study investigated the prevalence of elder abuse in a hospitalized population, and we found that one in six older adults had been subjected to elder abuse. Of those, a majority had also been subjected to abuse before the age of 65, and earlier life abuse was the only background factor significantly associated with subsequent elder abuse. Reporting victimization both before and after the age of 65 was associated with poor mental-health outcomes, while no association was found between mental ill-health and reporting only elder abuse or only earlier in life abuse. Altogether, our findings underline the importance of a life-course perspective in research on elder abuse.

### Prevalence of elder abuse

The prevalence of elder abuse in this hospitalized population was 18%. Hospital wards are a relatively unexplored setting for investigating elder abuse prevalence. There is one example of a study from India, which used a self-report measure and estimated a prevalence of 16%, which is similar to our findings [21]. Our reported prevalence is slightly higher compared to the 10%–15% often reported in community samples [5, 48, 49]. Hospitalized older adults often have risk factors for elder abuse such as high dependence on others for their daily living, and hence one could have expected an even higher prevalence compared to community samples. Why this was not the case could possibly be explained by a Neyman bias, whereby those who were most affected by disease were not included. Elder abuse has previously been associated with functional dependency as well as cognitive impairment. In this study, however, older adults who did not have the physical, cognitive, or linguistic capacity to fill out the REAGERA-S and participate in the interview were excluded. During the study period, 224 older adults (34%) admitted to the clinic were excluded for these reasons. It is plausible that the prevalence rate would have been higher if those older adults could have been included in the study. However, to include older adults with, e.g., cognitive impairment would require another study methodology.

It should also be noted that a prevalence of 10–15% in community samples often refers to victimization in the past year. In this study, we defined elder abuse as one or more abusive experiences after the age of 65. This definition is likely to lead to a higher prevalence compared to only past-year prevalence. Also, in meta-analyses and reviews on elder abuse, there is a great variation in estimated prevalence, even if data is limited to the past 12 months [5]. This could be a symptom of the methodological challenges in violence research, including elder abuse, where differences in definitions, operationalizations, and settings have a strong impact on the reported prevalence [10, 11, 50]. To be able to make comparisons between different populations, studies using the same methodology need to be carried out in different settings. However, our findings confirm that experiences of elder abuse are common among older adult hospitalized patients in Sweden.

The fact that victimization data was collected using a semi-structured qualitative interview rather than a questionnaire or structured interview could have affected the results in several ways. There was a dropout, whereby one in four who completed the instrument declined to participate in the interview. When validating the REAGERA-S, the non-interviewed group reported somewhat less abuse than the interviewed group [38]. This corresponds with previous research on non-response bias, where individuals who are not exposed are less inclined to participate in a study regarding the subject [51]. With this rationale, there might be a slight overestimation of prevalence in our results, due to the dropout rate. However, there should be a greater degree of certainty in the data, as the answers were validated against the participants in the interview, and misconceptions regarding questions could be clarified directly. It was also possible to address the challenge of defining abuse in the interviews. What constitutes an abusive experience must be defined partly by the victim, and the cultural norms and circumstances surrounding the abusive experience must be considered. As presented in the methods, this issue was handled systematically via discussions in the research group prior to classification.

### Background factors associated with elder abuse

Regarding predictors of elder abuse, previous studies have found divergent results concerning the associations between elder abuse and sex, age, and marital status, although a consistent association between elder abuse and functional dependency is reported [15, 16, 52]. It was hence unexpected that we did not find a significant association between reporting elder abuse and ADL status. This could potentially be explained by the rather low sample size, increasing the risk of a false negative. Hence, the lack of association with all background factors should be interpreted with some caution. However, we found that earlier life abuse increases the odds of reporting elder abuse, which is in line with previous research [15, 24, 26], and further underlines the importance of a life-course perspective in research on elder abuse. From a cumulative inequality perspective, it would have been interesting to differentiate the effect on subsequent elder abuse between earlier childhood abuse and other types of abuse, for example intimate partner violence and community violence. Cumulative inequality theory specifically states that childhood experiences play a special role in the development of a person’s life trajectory, and hence it would have been valuable to know whether it was uniquely childhood abuse that increased the odds of reporting elder abuse or whether previous adult victimization was also relevant. One Canadian study managed to make this differentiation, and found that only childhood abuse retained its effects after adjusting for other background variables [24]. This suggests that childhood abuse could, as cumulative inequality theory states, have a specific effect on vulnerability to elder abuse.

### Life-course victimization and mental ill-health

When exploring associations between abuse and mental ill-health, we found that only those reporting abusive experiences both before and after the age of 65 had increased odds of poor mental health. The associations were similar for all four outcomes, but were not significant in terms of having an anxiety or depression diagnosis. This indicates that a negative life trajectory with experiences of abuse earlier in life as well as in later life has the strongest impact on mental health. The difference in mental ill-health may also be attributed to the fact that all cases in that category were polyvictims, i.e., were victimized on several occasions or by several perpetrators. Polyvictimization has previously been reported to be associated with greater ill-health than any single victimization among older adults [37], and in this particular sample previous experiences of abuse were found to influence both the experience of elder abuse and how it was managed [39, 40]. It is therefore unfortunate that many studies on elder abuse focus on past-year exposure, and that polyvictimization is not further explored. Our finding that only the combination of victimization earlier in life and elder abuse was associated with mental ill-health indicates that there is a risk of disregarding substantial information when taking a narrow time perspective in the field of elder abuse. If only past-year victimization is considered, the association between abuse and mental ill-health may be misinterpreted, i.e., overestimated or underestimated due to disregarding previous life-course experiences of abuse.

As this was a cross-sectional study, causality cannot be assessed. The association between victimization and ill-health is complex, and the relationships may be bidirectional. Previously, childhood victimization has been found to be associated with ill-health in middle age, which in turn was associated with elder abuse [32]. There is also a well-established association between childhood abuse and adult intimate partner violence, and childhood victimization has been linked to later life mental ill-health [28, 53]. A few longitudinal studies have also been conducted in which abusive experiences in later life are concluded to be a risk factor for later mental ill-health, such as anxiety symptoms and major depression [4, 54]. Altogether, this supports the cumulative inequality theory, stipulating that abuse and ill-health aggravate each other throughout the life course.

### Clinical implications

Our finding that one in six patients had experienced elder abuse and the association found between life-course victimization and mental ill-health indicate that abusive experiences are important to consider in health care encounters. Previous studies have found that abusive experiences often go unnoticed in Swedish health care, and that many health care providers have never spoken to older patients about abuse [20, 55]. Hence, there is a need to increase the detection of elder abuse and earlier life experiences of abuse to improve the care given to older adults. One potential way forward could be to increase the use of screening instruments for detecting elder abuse, such as the REAGERA-S which also uses a life-course perspective [38].

### Limitations

One major limitation of this study was the rather small study sample, which was a consequence of using data previously collected for another purpose. Hence, the lack of significant associations between background characteristics and elder abuse (Table 2), as well as between some of the background characteristics and reporting the different forms of mental ill-health (Table 3), should be interpreted with caution. Also, because of the rather small sample size, all estimates come with wide confidence intervals and the odds ratios are less precise. However, despite the rather low number of participants, previous victimization was associated with elder abuse, and experiences of victimization both before and after 65 years were associated with different forms of ill-health. This finding indicates that a life-course perspective in research on elder abuse is important.

Data was only collected from internal medicine and geriatric wards, which limits the generalizability of the results. The prevalence rate might have been different had we also included patients from other settings, such as orthopedics or surgery. Also, as shown in Fig. 1, 38% (*n* = 115) of those asked to participate in the study (*n* = 306) declined and 24% (*n* = 46) of those who completed the first step by answering REAGERA-S (*n* = 191) did not participate in the interview. The total participation rate was 44% (*n* = 135/306), which is a limitation in terms of generalizability but is in line with previous research, e.g., a survey about elder abuse conducted in seven European countries, including Sweden, where the reported response rate was 45% [9]. Likewise, an overview of studies about intimate partner violence conducted in Sweden reported response rates in included studies ranging from 35 to 64% [50].

As previously mentioned, this was a cross-sectional study, and hence causality between background characteristics, abusive experiences, and mental health cannot be assessed. It should also be noted that the used measures of mental ill-health have some limitations. Diagnoses and medications do not necessarily mirror either the respondents’ level of suffering or their quality of life, and it would have been interesting to include measures of such aspects. Also, we did not consider protective factors for abuse or ill-health. Many previous studies on the relationship between victimization and mental ill-health highlight the impact and mediating effect of social support [4, 37], which we did not examine. Nor did we consider potential risk factors for elder abuse at societal level, such as family norms or organization of health care. For example, ageism is a factor at societal level which has been suggested as a risk factor for elder abuse that is rarely considered in studies [56, 57]. Future studies could consider this factor by measuring the effect of individual experiences of ageism, or ageist patterns in the older adult’s environment.

## Conclusions

We found that one in six older hospitalized patients had experiences of elder abuse, that earlier in life abuse was associated with elder abuse, and that the combination of earlier in life abuse and elder abuse was associated with mental ill-health. These findings indicate that a life-course perspective is important in research on elder abuse, as well as in clinical practice. The high prevalence of elder abuse among older hospitalized patients found in this study highlights that identifying and caring for victims of abuse should be a priority in hospital care of older adults.

## Supplementary Information

**Additional file 1:** **Supplementary file 1.** Interview guide.

## Data Availability

The datasets used and analyzed during the current study are available from the corresponding author upon reasonable request.

## Abbreviations

ADL: Activities of daily living
B-ADL: Basic activities of daily living
CI: Confidence interval
I-ADL: Instrumental activities of daily living
NaSSA: Noradrenaline and specific serotonergic antidepressants
OR: Odds ratio
PHQ-9: Patient health questionnaire-9
REAGERA-S: Responding to Elder Abuse in GERiAtric care – Self-administered
ROC: Receiver operating curve
SNRI: Serotonin and norepinephrine reuptake inhibitors
SSRI: Selective serotonin reuptake inhibitors

## References

[1] World Health Organization. World report on ageing and health. World Health Organization; 2015.

[2] World Health Organization (2002). The Toronto declaration on the global prevention of elder abuse.

[3] Yunus RM, Hairi NN, Choo WY (2019). Consequences of Elder Abuse and Neglect: A Systematic Review of Observational Studies. Trauma Violence Abuse.

[4] Acierno R, Hernandez-Tejada MA, Anetzberger GJ, Loew D, Muzzy W (2017). The National Elder Mistreatment Study: An 8-year longitudinal study of outcomes. J Elder Abuse Negl.

[5] Yon Y, Mikton CR, Gassoumis ZD, Wilber KH (2017). Elder abuse prevalence in community settings: a systematic review and meta-analysis. Lancet Glob Health.

[6] Ho CS, Wong SY, Chiu MM, Ho RC (2017). Global prevalence of elder abuse: a meta-analysis and meta-regression. East Asian Arch Psychiatry.

[7] Olofsson N, Lindqvist K, Danielsson I (2012). Fear of crime and psychological and physical abuse associated with ill health in a Swedish population aged 65–84 years. Public Health.

[8] Ahnlund P, Andersson T, Snellman F, Sundström M, Heimer G (2020). Prevalence and correlates of sexual, physical, and psychological violence against women and men of 60 to 74 years in Sweden. J Interpers Violence.

[9] Lindert J, de Luna J, Torres-Gonzales F, Barros H, Ioannidi-Kopolou E, Melchiorre MG (2013). Abuse and neglect of older persons in seven cities in seven countries in Europe: a cross-sectional community study. Int J Public Health.

[10] Sooryanarayana R, Choo W-Y, Hairi NN (2013). A Review on the Prevalence and Measurement of Elder Abuse in the Community. Trauma Violence Abuse.

[11] Dong XQ (2015). Elder Abuse: Systematic Review and Implications for Practice. J Am Geriatr Soc.

[12] Chatfield MD, Brayne CE, Matthews FE (2005). A systematic literature review of attrition between waves in longitudinal studies in the elderly shows a consistent pattern of dropout between differing studies. J Clin Epidemiol.

[13] Kelfve S, Thorslund M, Lennartsson C (2013). Sampling and non-response bias on health-outcomes in surveys of the oldest old. Eur J Ageing.

[14] Williams JL, Racette EH, Hernandez-Tejada MA, Acierno R. Prevalence of elder polyvictimization in the United States: data from the National Elder Mistreatment Study. J Interpers Violence. 2020;35(21–22):4517–32. 10.1177/0886260517715604. Epub 2017 Jun 23. 10.1177/0886260517715604 29294807

[15] Johannesen M, LoGiudice D (2013). Elder abuse: a systematic review of risk factors in community-dwelling elders. Age Ageing.

[16] Burnes D, Pillemer K, Caccamise PL, Mason A, Henderson CR, Berman J (2015). Prevalence of and Risk Factors for Elder Abuse and Neglect in the Community: A Population-Based Study. J Am Geriatr Soc.

[17] Yon Y, Ramiro-Gonzalez M, Mikton CR, Huber M, Sethi D (2019). The prevalence of elder abuse in institutional settings: a systematic review and meta-analysis. Eur J Pub Health.

[18] Dong X (2015). Screening for Elder Abuse in Healthcare Settings: Why Should We Care, and Is It a Missed Quality Indicator?. J Am Geriatr Soc.

[19] Swedish National Board of Health and Welfare. SOSFS 2014:4 Socialstyrelsens föreskrifter och allmänna råd om våld i nära relationer. [Regulations about violence in close relationships.] https://www.socialstyrelsen.se/globalassets/sharepoint-dokument/artikelkatalog/foreskrifter-och-allmanna-rad/2014-5-7.pdf.

[20] Simmons J, Swahnberg K (2022). Characteristics Associated With Being Asked About Violence Victimization in Health Care: A Swedish Random Population Study. J Interpers Violence.

[21] Nisha C, Manjaly S, Kiran P, Mathew B, Kasturi A (2016). Study on elder abuse and neglect among patients in a medical college hospital, Bangalore, India. J Elder Abuse Negl.

[22] Cohen M (2008). Research assessment of elder neglect and its risk factors in a hospital setting. Intern Med J.

[23] Post L, Page C, Conner T, Prokhorov A, Fang Y, Biroscak BJ (2010). Elder Abuse in Long-Term Care: Types, Patterns, and Risk Factors. Res Aging.

[24] McDonald L, Thomas C (2013). Elder abuse through a life course lens. Int Psychogeriatr.

[25] Asyraf M, Dunne MP, Hairi NN, Mohd Hairi F, Radzali N, Wan YC (2021). The association between elder abuse and childhood adversity: A study of older adults in Malaysia. PLoS ONE.

[26] Kong J, Easton SD (2019). Re-experiencing Violence Across the Life Course: Histories of Childhood Maltreatment and Elder Abuse Victimization. J Gerontol B Psychol Sci Soc Sci.

[27] Ferraro KF, Schafer MH, Wilkinson LR (2016). Childhood Disadvantage and Health Problems in Middle and Later Life: Early Imprints on Physical Health?. Am Sociol Rev.

[28] Maschi T, Baer J, Morrissey MB, Moreno C (2013). The Aftermath of Childhood Trauma on Late Life Mental and Physical Health: A Review of the Literature. Traumatology.

[29] Felitti VJ, Anda RF, Nordenberg D, Williamson DF, Spitz AM, Edwards V (1998). Relationship of childhood abuse and household dysfunction to many of the leading causes of death in adults. The Adverse Childhood Experiences (ACE) Study. Am J Prev Med.

[30] Ferraro KF, Shippee TP (2009). Aging and Cumulative Inequality: How Does Inequality Get Under the Skin?. Gerontologist.

[31] Ferraro KF, Shippee TP, Schafer MH. Cumulative inequality theory for research on aging and the life course. Handbook of theories of aging, 2nd ed. New York, NY, US: Springer Publishing Co; 2009. p. 413–33.

[32] Easton SD, Kong J (2021). Childhood adversities, midlife health, and elder abuse victimization: a longitudinal analysis based on cumulative disadvantage theory. J Gerontol B Psychol Sci Soc Sci.

[33] Turner HA, Finkelhor D, Ormrod R (2010). Poly-victimization in a national sample of children and youth. Am J Prev Med.

[34] Finkelhor D, Ormrod R, Turner H, Holt M (2009). Pathways to poly-victimization. Child Maltreat.

[35] Finkelhor D, Ormrod RK, Turner HA (2007). Poly-victimization: a neglected component in child victimization. Child Abuse Negl.

[36] Ramsey-Klawsnik H (2017). Older adults affected by polyvictimization: A review of early research. J Elder Abuse Negl.

[37] Simmons J, Swahnberg K (2021). Lifetime prevalence of polyvictimization among older adults in Sweden, associations with ill-heath, and the mediating effect of sense of coherence. BMC Geriatr.

[38] Simmons J, Wiklund N, Ludvigsson M, Nägga K, Swahnberg K (2020). Validation of REAGERA-S: a new self-administered instrument to identify elder abuse and lifetime experiences of abuse in hospitalized older adults. J Elder Abuse Negl.

[39] Ludvigsson M, Wiklund N, Swahnberg K, Simmons J (2022). Experiences of elder abuse: a qualitative study among victims in Sweden. BMC Geriatr.

[40] Simmons J, Wiklund N, Ludvigsson M (2022). Managing abusive experiences: a qualitative study among older adults in Sweden. BMC Geriatr.

[41] Bujang MA, Adnan TH (2016). Requirements for Minimum Sample Size for Sensitivity and Specificity Analysis Journal of clinical and diagnostic research. JCDR.

[42] Kroenke K, Spitzer RL, Williams JB (2001). The PHQ-9: validity of a brief depression severity measure. J Gen Intern Med.

[43] Tavakol M, Dennick R (2011). Making sense of Cronbach's alpha. Int J Med Educ.

[44] Kroenke K (2003). Patients presenting with somatic complaints: epidemiology, psychiatric co-morbidity and management. Int J Methods Psychiatr Res.

[45] Haug TT, Mykletun A, Dahl AA (2004). The Association Between Anxiety, Depression, and Somatic Symptoms in a Large Population: The HUNT-II Study. Psychosom Med.

[46] Drayer RA, Mulsant BH, Lenze EJ, Rollman BL, Dew MA, Kelleher K (2005). Somatic symptoms of depression in elderly patients with medical comorbidities. Int J Geriatr Psychiatry.

[47] Hosmer DW, Loemeshow S. Assessing the Fit of the Model. Applied Logistic Regression. 2nd ed. New York: Wiley; 2000. p. 143–202.

[48] Acierno R, Hernandez MA, Amstadter AB, Resnick HS, Steve K, Muzzy W (2010). Prevalence and correlates of emotional, physical, sexual, and financial abuse and potential neglect in the United States: the National Elder Mistreatment Study. Am J Public Health.

[49] Du P, Chen Y (2021). Prevalence of elder abuse and victim-related risk factors during the COVID-19 pandemic in China. BMC Public Health.

[50] Simmons J, Swahnberg K (2019). Can nonresponse bias and known methodological differences explain the large discrepancies in the reported prevalence rate of violence found in Swedish studies?. PLoS ONE.

[51] Galea S, Tracy M (2007). Participation Rates in Epidemiologic Studies. Ann Epidemiol.

[52] Pillemer K, Burnes D, Riffin C, Lachs MS (2016). Elder Abuse: Global Situation, Risk Factors, and Prevention Strategies. The Gerontologist.

[53] Easton SD, Kong J, Gregas MC, Shen C, Shafer K (2019). Child Sexual Abuse and Depression in Late Life for Men: A Population-Based, Longitudinal Analysis. J Gerontol B Psychol Sci Soc Sci.

[54] Wong JS, Waite LJ (2017). Elder mistreatment predicts later physical and psychological health: Results from a national longitudinal study. J Elder Abuse Negl.

[55] Motamedi A, Ludvigsson M, Simmons J (2022). Factors associated with health care providers speaking with older patients about being subjected to abuse. J Elder Abuse Negl.

[56] Pillemer K, Burnes D, MacNeil A (2021). Investigating the connection between ageism and elder mistreatment. Nature Aging.

[57] Storey JE (2020). Risk factors for elder abuse and neglect: A review of the literature. Aggress Violent Beh.

