# Supplementary material for: Elder abuse and life-course victimization in hospitalized older adults in Sweden: prevalence and associations with mental ill-health
Source: BMC Geriatr. 2022 Dec 2;22:929. doi: 10.1186/s12877-022-03638-8 (PMC9716666; doi:10.1186/s12877-022-03638-8)
Supplement: Supplementary file 1 — Additional file 1: Supplementary file 1. Interview guide. [file 12877_2022_3638_MOESM1_ESM.pdf]

Supplementary file 1. Interview guide.

Part 1. Background questions

1. What is your level of education?
    - 6-7 years
    - 8-9 years
    - 10-12 years
    - 13 years or more

2. What is your current marital status?
    - Married/living together with partner
    - Unmarried
    - Divorced
    - Widow/widower

3. Do you have children?
    - Yes
    - No

4. Do you live together with anyone?
    - Living alone
    - Living with partner/husband/wife
    - Living together with other person

5. What is your housing situation?
    - House or apartment
    - Assisted living

6. Do you have anyone to help you get groceries, cook, do laundry or clean?
    - No
    - Yes, family member
    - Yes, cleaning company or home care staff

7. Does anyone help you with your medications?
    - No
    - Yes, family member
    - Yes, home care staff or other health care professional
    - I do not use any medication

8. Does anyone help you with your daily needs such as personal hygiene, get dressed, use the bathroom etc?
    - No
    - Yes, family member
    - Yes, home care staff or other health care professional

9. Do you use any mobility aids?
    - No
    - Yes, walker, cane or crutch
    - Yes, wheelchair

Part 2. Questions about abuse

It is relatively common for older adults to be exposed to negative events. These can be physical, psychological or sexual abuse or violations, but can also involve being exploited financially or if someone refuses to help you even though you need help to cope with daily living. They could be things that happened a long time ago or recently. Have you experienced anything like that?

☐ Yes, before the age of 65 ☐ Yes, after the age of 65 ☐ No, never

General exploratory questions

- Has anyone been mean to you? Has anyone treated you badly?
- Has anyone talked to you in a way that made you feel bad or upset you?
- Have you ever had a relationship with someone who has not been kind to you? What about your relationship with your children, spouse, neighbors?
- Is there anyone that you feel uncomfortable with? Or afraid of?

Physical violence

☐ Yes, before the age of 65 ☐ Yes, after the age of 65 ☐ No, never

- Have you ever been hit/beaten or exposed to some other act that could have hurt you?

Psychological/emotional violence

☐ Yes, before the age of 65 ☐ Yes, after the age of 65 ☐ No, never

- Have you ever felt like someone tried to control you or humiliated you?
- Has anyone forced you to do things you did not want to do? What kind of things? How were you forced?
- Have you ever been threatened?

Sexual violence

☐ Yes, before the age of 65 ☐ Yes, after the age of 65 ☐ No, never

- Has anyone touched you in a way that didn't feel right?
- Have you been forced into sexual acts?

Economical violence

☐ Yes, before the age of 65 ☐ Yes, after the age of 65 ☐ No, never

- Have you been cheated out of money? Cheated/forced to sign financial papers (that you did not know the content of)?
- Have you been robbed of money or other valuables (by next of kin or a member of staff)?

Neglect

☐ Yes, before the age of 65 ☐ Yes, after the age of 65 ☐ No, never

- When staff have helped you, has anyone spoken condescendingly to you? Or has anyone done something that did not feel good or made you feel bad or offended? Hurt you consciously or unconsciously?
- Have you ever not received the help you needed?

Childhood abuse: ☐ Yes ☐ No ☐ By a family member ☐ By another perpetrator

Intimate partner violence: ☐ Yes ☐ No

If yes to any question: Do you suffer as a result of your experiences? Have they affected you in any way?

☐ Yes ☐ No

Did you fill out the questionnaire yourself? ☐ Yes ☐ No

Note: Whenever abusive experiences were revealed, a qualitative interview was conducted with more explorative questions.
